# Progress on the HIF-1α/VEGF/VEGFR2 signal pathway in hepatic alveolar echinococcosis

**DOI:** 10.3389/fonc.2025.1553125

**Published:** 2025-04-08

**Authors:** Meng-Zhao Xu, Fei Ke, Jin-Ping Chai, Ji-De A, Qing-Long Tan

**Affiliations:** ^1^ The Graduate School, Qinghai University, Xining, China; ^2^ Department of Internal Medicine-Cardiovascular, Qinghai Provincial People’s Hospital, Xining, China; ^3^ Department of Hepatic Hydatidosis, Qinghai Provincial People’s Hospital, Xining, China; ^4^ Department of General Surgery, Qinghai Provincial People’s Hospital, Xining, China

**Keywords:** hepatic alveolar echinococcosis, signal pathway, hypoxia-inducible factor-1α, vascular endothelial growth factor, vascular endothelial growth factor receptor 2, angiogenesis

## Abstract

Alveolar echinococcosis (AE), a lethal parasitic zoonosis mimicking malignant tumors, progresses via hepatic infiltration and metastatic spread, causing multiorgan failure. Despite its clinical resemblance to cancer, molecular drivers of its aggressiveness remain poorly defined. Recent studies highlight perilesional angiogenesis as pivotal for lesion invasiveness, mediated by VEGF-driven pathological vascularization. VEGF not only fuels parasitic proliferation by creating nutrient-rich microenvironments but also engages crosstalk with host-parasite interactions, including immune evasion by Echinococcus multilocularis, germinal layer hyperplasia, and periparasitic inflammation.Targeting the HIF-1α/VEGF/VEGFR2 axis emerges as a promising therapeutic strategy. Mechanistically, VEGF/VEGFR2 blockade may simultaneously disrupt angiogenesis-dependent parasitic expansion and survival pathways. Preclinical evidence shows that inhibiting HIF-1α (VEGF’s upstream regulator) suppresses metacestode proliferation and tissue invasion by starving lesions of vascular support while modulating immune-inflammatory responses. This dual action addresses both parasitic resource acquisition and host defence subversion.This review synthesizes molecular insights into HIF-1α/VEGF-mediated pathogenesis with clinical observations, proposing anti-angiogenic therapy as a rational adjunct to current treatments. By delineating VEGF’s role in sustaining parasitic metabolic demands and immune regulation, we underscore the translational potential of pathway-specific inhibitors. Such approaches could mitigate limitations of conventional therapies (e.g., benzimidazoles), particularly for advanced-stage AE with microvascular proliferation. Systematic analysis of angiogenesis signalling networks advances our understanding of AE’s “parasitic cancer” paradigm while guiding development of targeted interventions to improve patient outcomes.

## 1 Introduction

Hepatic alveolar echinococcosis (HAE) is a parasitic disease caused by the infection of the liver by *Echinococcus multilocularis* larvae, transmitted through the accidental ingestion of contaminated food and water by both animals and humans. The eggs hatch in the digestive tract, invade the intestinal wall, enter the portal system through blood circulation, and eventually colonize the liver and other organs. This disease severely impacts the life and health of people in agricultural and pastoral areas and hinders the region’s economic development, making it one of the four most important infectious diseases in pastoral areas (1–3). In China, HAE is most prevalent in Xinjiang, Qinghai, Tibet, Ningxia, Gansu, and Sichuan provinces (autonomous regions) (4). HAE has an insidious onset and long latency period, with most patients showing no early symptoms. By the time it is detected, the disease is already in an advanced stage and may have metastasized to distant organs, such as the brain, lungs, and bones, earning it the nickname “second liver cancer” (5, 6). Pathological vascular proliferation is an important feature of most malignant tumours, and neovascularisation facilitates tumour invasion and metastasis. Studies have shown significant neovascularisation around HAE foci, which may be crucial for its infiltrative growth and distant metastasis (7). Recent research has highlighted the role of the HIF-1α/VEGF/VEGFR2 signalling pathway in HAE pathogenesis (8). As an important mediator of angiogenesis, VEGF and its mediated downstream signalling pathway factors promote HAE angiogenesis to accelerate the proliferation and invasion of lesions. Animal studies have shown that various VEGF and VEGF receptor inhibitors can target VEGF or VEGFR2, thereby blocking the VEGF/VEGFR2 pathway and its mediated downstream effects, ultimately exerting an antivascular effect on HAE (9, 10). This article discusses the specific role of the HIF-1α/VEGF/VEGFR2 pathway in HAE pathogenesis and the current research status of VEGF-related inhibitors in HAE.

## 2 Overview of the HIF-1α/VEGF/VEGFR2 signalling pathway and its role in liver diseases

Hypoxia-inducible factor-1 (HIF-1) is a heterodimeric transcriptional activator consisting of two subunits, α and β. The β subunit belongs to the family of nuclear transporter proteins of the aromatic hydrocarbon receptor, whose expression is unaffected by the concentration of oxygen, contributing to the stability of HIF-1 in the nucleus and its conformational transition after dimerization. The α subunit has three isoforms, HIF-1α, 2α, and 3α, encoding 826, 869, and 668 amino acids, respectively (11, 12). HIF-1α, HIF-2α, and HIF-3α are differentially expressed in different tissues. For example, HIF-1α is constitutively expressed in most biological tissues, HIF-2α is selectively expressed in cells such as hepatocytes and endothelial cells, and HIF-3α is mainly expressed in the heart, skeletal muscle, and placenta (13). Currently, HIF-1α plays an important role in tumour angiogenesis, and the oxygen content of the tissue microenvironment regulates its expression. Under normoxic conditions, the proline residue of HIF-1α is hydroxylated by oxygen-dependent prolyl hydroxylase, which then binds to the von Hippel- Lindau tumour suppressor protein (pVHL), a protein that has the function of targeting HIF-1α degradation, leading to the ubiquitinated degradation of HIF-1α. Subsequent binding to pVHL, which targets HIF-1α degradation, ultimately leads to ubiquitinated degradation of HIF-1α. Conversely, when the tissue microenvironment is in a state of continuous hypoxia, it causes the release of HIF-1α, which combines with HIF-1β to form the HIF-1 complex. This complex enters the nucleus and binds to the HIF response element, promoting the transcription of downstream genes, such as VEGF and glucose metabolism-related genes, and thereby regulating physiological processes such as cell metabolism and angiogenesis. Moreover, it regulates a variety of angiogenic factors, including VEGF, platelet-derived growth factor(PDGF), fibroblast growth factor(FGF), and angiopoietin, which promote their release from endothelial cells and stroma cells. Meanwhile, the expression of HIF-1α promotes the differentiation of endothelial progenitor cells to mature endothelial cells, which recruits endothelial cells and promotes angiogenesis. Additionally, it attracts promoter factors that promote the differentiation of medullary cells into tumour-associated macrophages or neutrophils, further promoting angiogenesis (14, 15).

**Figure 1:**
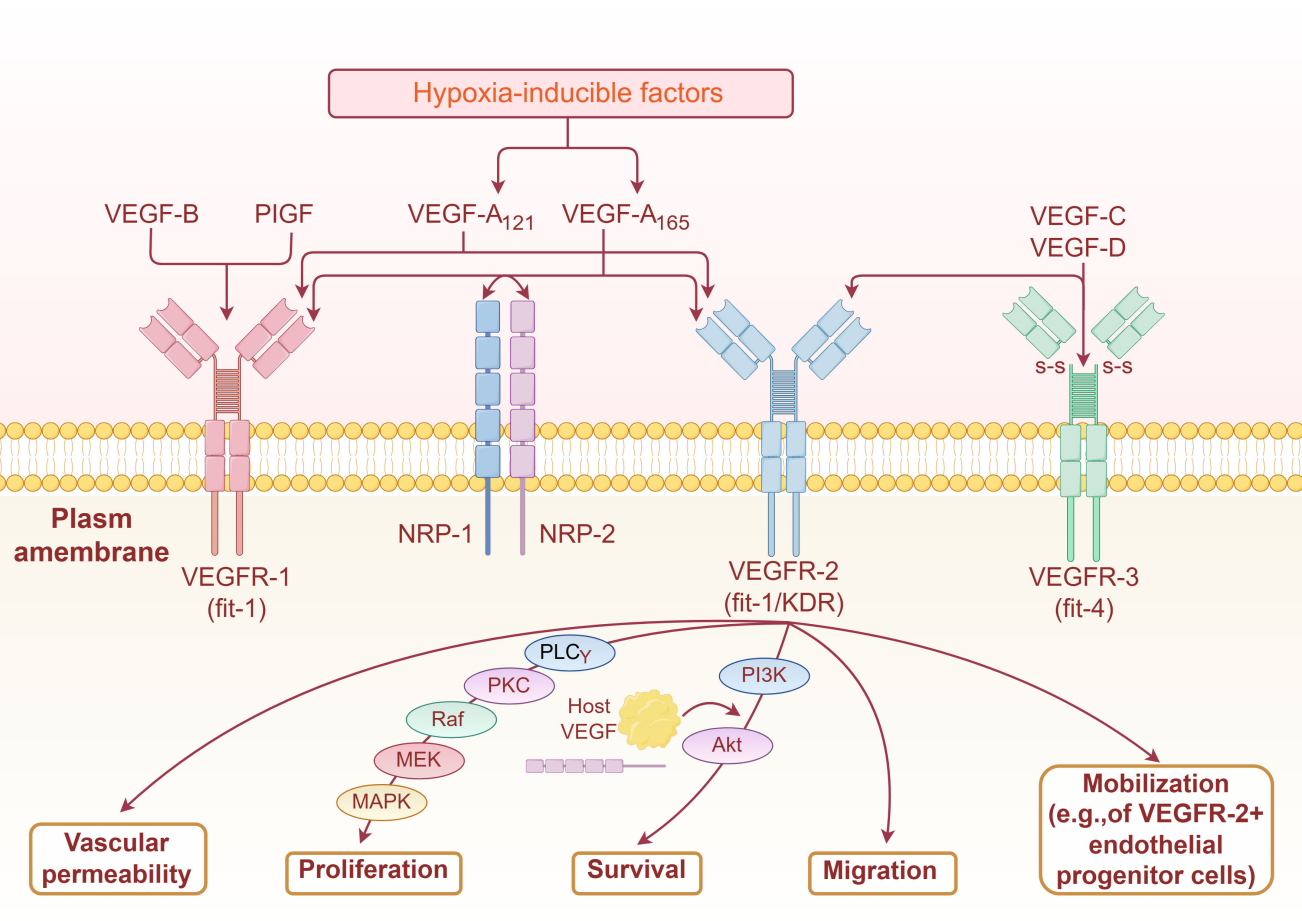
Mechanism of VEGF signaling pathway in stimulating sngiogenesis (By Figdraw2.0).

VEGF, also known as vascular permeability factor, is a dimeric glycoprotein consisting of 165 amino acid residues that specifically promote the growth and proliferation of vascular endothelial cells. VEGF can promote an increase in vascular permeability, extracellular matrix denaturation, vascular endothelial cell migration, proliferation, and angiogenesis. In mammals, there are seven VEGF families: VEGFA, VEGFB, VEGFC, VEGFD, VEGFE, snake venom VEGF (snake venom VEGF, sv-VEGF), and placental growth factor (placentrowthactor, PIGF). VEGF usually refers to VEGFA, which contains five isoforms: VEGFA121, VEGFA145, VEGFA165, VEGFA189, and VEGFA206.VEGFA165 is the major isoform of VEGF-A, playing a central functional role in all VEGF isoforms (16, 17). High-affinity receptors that bind specifically to VEGF are called vascular endothelial growth factor receptors (VEGFR) and include transmembrane tyrosine kinase receptors (VEGFR-1, VEGFR-2, and VEGFR-3) and non-tyrosine kinase receptors (NPR-1 and NPR-2) (18). The cell surface receptors involved in the VEGF/VEGFR pathway are mainly VEGFR2 receptors. The VEGF/VEGFR2 signalling pathway is as follows: VEGF binds to the immunoglobulin (Ig)-like domains on the extracellular segments of VEGFR2 and induces dimerization of VEGFR2, which triggers phosphorylation of tyrosine sites on the intracellular segments. Phosphorylation of tyrosine residues in the intracellular segment activates downstream signalling molecules to promote vascular endothelial cell proliferation and increase vascular permeability (17, 18) (**Figure 1**).

As a classical signalling pathway that is widely present in eukaryotes and has a vast range of roles, the HIF-1α/VEGF/VEGFR2 signalling pathway is well-known in liver diseases, and it can regulate the expression of related genes and plays an important role in liver-related malignancies, cirrhosis, inflammation, and other diseases (19). For example, Michael A et al. demonstrated the important role of VEGF-mediated angiogenesis in hepatocellular carcinoma, which is a potential molecular target for hepatocellular carcinoma treatment and prognostic evaluation (20). Coulon S et al. demonstrated that blockade of VEGFR2 attenuates steatosis and inflammation in a diet-induced NASH mouse model through the establishment of an animal model (21). The VEGF pathway was confirmed to play a key role in the progression of non-alcoholic steatohepatitis. In HAE, studies on mice infected with *Echinococcus multilocularis* revealed differences in HIF-1α/VEGF expression levels in echinococcal foci, the tissue adjacent to the foci, and the normal liver tissue. This suggests that VEGF is a key mediator of tissue neovascularisation in the infiltration zones around the foci of HAE (9). Studying the expression and functional identification of genes and proteins related to the VEGF signalling pathway in echinococcosis can help understand the progression of HAE at the cellular and molecular levels, reveal the relationship between Echinococcus and the human body in various types of pathological processes, and provide a relevant scientific basis for the prevention and treatment, prognosis, and other aspects of HAE.

## 3 The HIF-1α/VEGF/VEGFR2 signalling pathway induces angiogenesis and contributes to the pathogenesis of hepatic alveolar echinococcosis

Angiogenesis was often neglected in early studies of HAE. However, with more in-depth research, its role in disease progression has received increasing attention. The HIF-1α/VEGF/VEGFR2 signalling pathway, a key angiogenic mediator, plays an important role in the occurrence and development of HAE. VEGF regulates vascularisation through the activation of downstream signalling factors. Abnormal differentiation of endothelial cells promotes angiogenesis, alters vascular permeability, and accelerates worm growth, invasion, and metastasis of HAE lesions (22).

### 3.1 The characteristics of blood supply for HAE

Microvessel density (MVD) is a reliable quantitative index reflecting the degree of tumour vascularisation. MVD can indicate whether a malignant tumour is invasive and metastatic and is crucial in determining prognosis (23). Neovascularisation, the sprouting of new blood vessels from existing ones, is essential for solid tumours’ initiation, growth, and metastatic spread. The vascular network within the tumour promotes nutrients, oxygen, and immune cell transport and is regulated by pro-angiogenic and anti-angiogenic factors (24). The proliferation and metastasis of HAE depend on significant neovascularisation around lesions, comparable to hepatic malignant tumours; HAE lesions have a large amount of vascular proliferation (25). Pathological observation shows abundant neovascularisation, fibrous connective tissue proliferation, and infiltration of eosinophils, lymphocytes, plasma cells, and multinucleated giant cells around the cystic wall of HAE lesions. This rich microvascular environment promotes blood vessel growth and lesion metastasis. A rich micro-blood supply enhances nutrients and oxygen absorption from the internal environment by the outer stratum corneum through the capillary network, accelerating the propagation of original head nodules and promoting lesion proliferation (26).

Numerous imaging and histological studies have reported substantial neovascularisation around HAE lesion tissues. Song T et al. (27) used ultrasonography and animal experiments to obtain the ultrasonographic intensity of the infiltration and proliferation zone around the HAE lesion. Combined with the microvessel density and other indicators, they confirmed a rich vascular supply in the infiltration and proliferation zone next to the HAE lesion through a correlation study and explained the pathological characteristics, clarifying the conditions for lesion invasion and metastasis. The pathological characteristics of the proliferative zone clarified that vascular proliferation next to HAE lesions provides necessary conditions for lesion invasion and metastasis. Yang J et al. (28) studied the infiltration range and blood flow status using Computed Tomography(CT) perfusion imaging and confirmed higher blood perfusion in the peripheral infiltration zones of human HAE larval tissues compared to those inside the lesion and surrounding liver tissues. Jiang Y et al. (29) used dual-source dual-energy CT (DSDECT) to evaluate the distribution of blood supply in HAE lesions. Their findings indicated a higher level of perfusion at the edge of HAE lesions, suggesting a vascular distribution in the edge region of these lesions. MVD counts increased, reflecting increased local perfusion and vascular permeability. HAE lesions had higher iodine values, and MVD counts in the edge region, indicating an increase in the blood supply and vascular permeability in this area. The iodine value and MVD were higher in the border region. The iodine value was significantly correlated with MVD, and this positive correlation was only observed in the border region of HAE lesions. No correlation exists between iodine value and MVD in the solid part of the lesions. MVD influenced the degree of enhancement of the lesions on DSDECT, and the region with the greatest enhancement was often accompanied by the highest MVD, indicating that the degree of enhancement reflected the characteristics of microvasculature distribution of the HAE lesions.

These findings indicate that the proliferative infiltration area adjacent to the HAE lesion is actively angiogenic, and these neovascularisations provide an essential basis for further proliferation and metastasis of the lesion, which can indirectly respond to the growth of the worms and biological information. The study of its blood supply status and the clarification of its growth and degree of infiltration can help deepen the understanding of the development of HAE lesions and indirectly assess the activity of HAE lesions, therefore guiding the treatment.

### 3.2 VEGF-induced angiogenesis promotes invasive growth of HAE lesions

VEGF has been proven to be closely related to infiltration, metastasis, disease progression, and prognosis of various solid tumours such as hepatocellular carcinoma, lung cancer, and breast cancer. VEGF overexpression has been observed in various malignant tumours. Studies have shown that compared to benign liver lesions such as hepatic haemangiomas and hepatic cysts, patients with primary hepatic malignant tumours have significantly higher serum VEGF expression levels, which often indicate a poor prognosis (30). Animal experiments showed that VEGF overexpression occurred around HAE lesions. Jiang HJ et al. (9, 22) established a mouse model of HAE and investigated vascularization in Echinococcus multilocularis lesions via immunohistochemical staining. Western blotting was employed to analyse the expression levels of HIF-1α, VEGFA, and VEGFR2 in perilesional infiltrative zone tissue, lesional tissue, and normal liver tissue. The study revealed abundant neovascularization surrounding the lesions in experimental mice, accompanied by significantly upregulated expression of VEGFA and VEGFR2, with a significant positive correlation between their expression levels.

VEGF plays several key roles in the invasive growth and metastasis of malignant tumours: VEGF secreted by tumour cells specifically binds to three different tyrosine kinase receptors (VEGFR1/2/3) in vascular endothelial cells and phosphorylates tyrosine residues in the intracellular domains after the formation of the dimer. The phosphorylated tyrosine residues activate the downstream signalling pathway proteins and related enzymes, which act directly on the vascular endothelial cells, promoting endothelial cell proliferation, migration, survival, increased permeability, and stimulating vascular growth in tumour tissues (31); VEGF induces endothelial cell activation, including changes in endothelial cell morphology, cytoskeletal alterations, migration, and growth. VEGF increases the expression of various endothelial cell genes, such as procoagulant tissue factor, fibrinolytic pathway protein, urokinase, tissue-type fibrinogen activator, type 1 fibrinogen activator inhibitor, urokinase inhibitor, and various mitogens (32, 33). Changes in VEGF levels affect the fibrinogen system, promoting the expression of the uPA receptor and tissue-type plasminogen activator in vascular endothelial cells. This results in the destruction of vascular extracellular matrix components, increased vascular permeability, and the creation of favourable conditions for the migration of vascular endothelial cells (34); VEGF can induce the release of nitric oxide to promote vasodilation and increase blood flow (35). An important interaction exists between VEGF and vasoregulatory immune cells, which can function as immunomodulatory factors within the tumour microenvironment. Under hypoxic conditions, upregulated VEGF is essential for the induction of immunosuppressive cells and regulates the function of T cells (effector T cells, regulatory T cells) and myeloid cells (dendritic cells, tumour-associated macrophages, and myeloid-derived suppressor cells) to promote tumour progression in a VEGF receptor-mediated manner. A few of these immune cells orchestrate tumour angiogenesis by directly determining the endothelial phenotype and function in the tumour vasculature through the release of pro-angiogenic or anti-angiogenic chemicals or indirectly by intercellular signalling and polarising other immune cells to exhibit inhibitory or regulatory properties (36, 37).

## 4 Role of VEGF downstream signalling effectors in the pathogenesis of HAE

### 4.1 MAPK signalling pathway

The mitogen-activated protein kinase (MAPK)signaling pathway is a highly conserved intracellular signal transduction system that regulates diverse biological processes, including cell proliferation, differentiation, apoptosis, stress responses, and inflammation (38).Activation of this pathway occurs through a three-tiered phosphorylation cascade: upstream MAP kinase kinase kinases (e.g., Ras proteins) phosphorylate and activate MAP kinase kinases, which subsequently phosphorylate MAP kinases (MAPKs). The activated MAPKs then translocate to the nucleus to mediate transcriptional responses to extracellular stimuli (39). Based on structural and functional characteristics, the MAPK family is categorized into four major subfamilies: the extracellular signal-regulated kinase 1/2 (ERK1/2) pathway, the c-Jun N-terminal kinase (JNK) pathway, the p38 MAPK pathway (40).

In AE, *Echinococcus multilocularis* protoscoleces activate ERK kinases in rat hepatocytes and exhibit partial activation of JNK and p38 kinases. Furthermore, these protoscoleces also induce ERK phosphorylation in hepatocellular carcinoma cells. These findings suggest that specific cytokines secreted by E. multilocularis protoscoleces, such as EmIns and EmBMP1/2, may bind to host surface receptors, thereby triggering MAPK signaling pathway activation in host hepatocytes (41). Mechanistically, proliferation of host hepatocytes is associated with infection-induced ERK1/2 pathway activation. Activated ERK upregulates matrix metalloproteinases (MMPs, e.g., MMP-2 and MMP-9), which degrade extracellular matrix (ECM) components, thereby facilitating invasive growth of hydatid cysts. In contrast, hepatocyte apoptosis is primarily mediated by E. multilocularis-stimulated JNK pathway activation. Conversely, p38 MAPK pathway activation may attenuate inflammatory responses during infection (42, 43).Activation of the MAPK pathway also plays a pivotal role in regulating macrophage functional polarization. Studies demonstrate that Echinococcus multilocularis soluble antigens activate RhoA, a member of the small GTPase family, which subsequently triggers phosphorylation of Raf—a critical upstream effector of the MAPK cascade—thereby enhancing MAPK signaling (44). Upon host exposure to E. multilocularis antigens, upregulated expression of RhoA and its downstream targets (Rho-associated kinase 1/2, ROCK1/2), along with MAPK-associated proteins including Tau, phosphorylated mitogen-activated protein kinase 3 (p-MAPK3), apoptosis signal-regulating kinase 1 (ASK1), and activating transcription factor 2 (ATF2), promotes macrophage polarization toward the M2 phenotype (45). M2-polarized macrophages secrete TGF-α and TGF-β, which stimulate hepatic stellate cell (HSC) proliferation and transdifferentiation, thereby enhancing collagen gene transcription and driving hepatic fibrogenesis (46, 47)(**Figure 1**).

### 4.2 PI3K/AKT/mTOR signaling pathway

The PI3K/AKT/mTOR signaling pathway, extensively studied in various cancers, regulates tumour cell growth, migration, and drug resistance. PI3K (phosphatidylinositol 3-kinase) is an intracellular lipid kinase composed of a regulatory subunit (p85) and a catalytic subunit (p110). Upon binding to growth factor receptors, PI3K activates AKT (protein kinase B), which subsequently modulates cellular metabolism, growth, proliferation, and survival (48). Activated AKT translocates to the cytoplasm or nucleus to phosphorylate downstream effector mTOR (mechanistic target of rapamycin), thereby initiating the PI3K/AKT/mTOR signaling cascade (49). In alveolar echinococcosis (AE), Zhang et al. demonstrated through a murine infection model that Echinococcus multilocularis protoscoleces (PSCs) enhance glycolysis via the PI3K/Akt/mTOR pathway to promote M2 macrophage polarization, ultimately accelerating AE progression (50) (**Figure 1**).

## 5 VEGF pathway inhibitors in the treatment of HAE

The treatment of HAE is primarily based on surgery; however, most patients with multi-organ metastasis at the onset of the disease and are no longer eligible for surgery. Additionally, there is no drug available that can eradicate AE. The clinical therapeutic drugs recommended by the WHO can only inhibit the disease, making radical treatment difficult to achieve. After decades of continuous use, albendazole, mebendazole, and other drug-resistance problems in the treatment of AE have become increasingly serious, and long-term medication has had a severe impact on liver and kidney function (51). Animal experiments have shown that VEGF inhibitors are effective in treating HAE. Existing VEGF inhibitors mainly include 3 major classes: VEGF monoclonal antibodies (e.g., bevacizumab and ranibizumab), VEGFR2 antagonists (e.g., ramucirumab), and tyrosine kinase inhibitors (e.g., sunitinib and sorafenib) (52).

VEGF monoclonal antibody inhibits the VEGF/VEGFR2 signalling pathway by blocking VEGF and preventing the phosphorylation of VEGFR2. Therefore, targeting VEGF signalling may be beneficial for treating HAE. Bevacizumab, a synthetic IgG1-type monoclonal antibody against VEGF, specifically binds to VEGF receptor signalling in endothelial cells. Bevacizumab blocks the MAPK/ERK pathway, inhibits the anti-apoptotic effect of VEGF, promotes apoptosis, destroys neovascularised endothelial cells, and blocks nutrient supply to the limbic zone, thereby exerting an inhibitory effect on HAE without affecting normal blood vessels or hepatocytes (53). In an experiment by Sang ZJ et al. mice infected with vesicular Echinococcus larvae were treated with bevacizumab through hepatic artery infusion. In the experimental group, structural malfunctions of vesicular Echinococcus larvae can be observed under light microscopy, including denaturation, separation or fracture of the cuticle and germinal layer, detachment, thinning of the cortex, destruction of part of the structure, deformation of the original head joints in the vesicle, and disintegration. Although bevacizumab destroys the HAE cells themselves, it destroys the microenvironment on which HAE relies, thereby inhibiting the rapid growth of HAE (54). However, the use of bevacizumab alone cannot eradicate HAE; it only slows down the growth rate. Therefore, the next focus of the research will be on the combination of albendazole treatment.

Sorafenib is an anticancer drug approved by the FDA for the treatment of unresectable hepatocellular carcinoma and advanced renal cell carcinoma. Sorafenib inhibits tumour growth and angiogenesis by targeting the RAF/MEK/ERK pathway and receptor tyrosine kinases (55). Dang ZS (56) et al. established a mouse model of HAE through animal experiments. After treatment with sorafenib (tyrosine kinase inhibitor), the cells in the inner hair-growth layer of the lesion capsule were detached and completely inactivated, showing superior therapeutic effects compared to the albendazole-treated group. Jiang H (57) et al. used sunitinib malate for the treatment of mouse animal models infected with vesicular bulbous larvae. They utilized WB, reverse transcription-quantitative polymerase chain reaction (RT-qPCR), and ELISA to detect the expression levels of extracellular VEGFA, VEGFR2, and phosphorylated VEGFR2 (p-VEGFR2) in hepatocytes. They observed that sunitinib malate treatment resulted in a significant decrease in the expression of VEGFA, VEGFR2, and p-VEGFR2. VEGFR2 expression was significantly reduced, which led to a significant reduction in neovascular lesion formation and inhibited the growth of HAE lesions in mice. This report demonstrates the potential effectiveness of blocking the VEGF pathway for the treatment of HAE. However, further investigation is required to assess the efficacy of sorafenib and other anti-VEGF agents in treating patients with HAE.

VEGF pathway inhibitors represent a promising therapeutic strategy for AE; however, their clinical application faces challenges including mechanistic complexity, safety concerns, and insufficient clinical evidence. Future research should integrate interdisciplinary approaches to elucidate the host-parasite interaction network, optimize combinatorial therapies, and leverage technological innovations to facilitate clinical translation. With advancements in precision medicine and policy support for rare disease research, VEGF-targeted therapies are poised to become an integral component of comprehensive AE management.

## 6 Summary and outlook

However, the pathogenesis of HAE is not fully understood. Imaging and histological studies have shown that angiogenesis is crucial in the progression of HAE; however, the molecular mechanisms related to angiogenesis are not well understood. The HIF-1α/VEGF/VEGFR2 pathway, as a classical angiogenic signaling pathway plays a vital role in the disease progression of HAE. VEGF is crucial in HAE because it is bonded to VEGFR2 and activates downstream signaling factors that mediate angiogenesis around HAE lesions and promote proliferation, invasion, and metastasis. By binding to VEGFR2, VEGF activates downstream signaling factors that mediate periportal angiogenesis in HAE lesions to promote lesion proliferation, invasion, and metastasis. Therefore, an in-depth study of the HIF-1α/VEGF/VEGFR2 signaling pathway can help to elucidate the mechanism of action of HAE, which in turn may lead to the development of more efficacious therapeutic drugs for HAE. VEGF inhibitors are a potential novel drug for the treatment of HAE, pointing to a new direction in its management. In animal experiments, VEGF inhibitors have shown better therapeutic effects in treating HAE. With the continuous in-depth research on the HIF-1α/VEGF/VEGFR2 signaling pathway in the mechanism of HAE, it is expected that VEGF inhibitors will be applied in the clinical treatment of HAE soon.
